# Potential Drug Dose‐Specific Adverse Three‐Drug Combinations: A US Insurance Claims Data‐Based Study

**DOI:** 10.1002/pds.70199

**Published:** 2025-08-17

**Authors:** Y. Shi, A. Sun, C. W. Chiang, Y. Yang, K. M. Hunold, J. Xu, M. Russo, J. Caterino, M. T. Eadon, L. Li, J. Su, M. Donneyong, P. Zhang

**Affiliations:** ^1^ Department of Biostatistics and Health Data Science Indiana University Indianapolis Indiana USA; ^2^ Department of Biomedical Informatics The Ohio State University Columbus Ohio USA; ^3^ Department of Emergency Medicine The Ohio State University Columbus Ohio USA; ^4^ Department of Statistics The Ohio State University Columbus Ohio USA; ^5^ Department of Internal Medicine The Ohio State University Columbus Ohio USA; ^6^ Department of Medicine Indiana University School of Medicine Indianapolis Indiana USA; ^7^ College of Pharmacy The Ohio State University Columbus Ohio USA

**Keywords:** adverse drug events, dose, drug combination, pharmacoepidemiology, toxicity

## Abstract

**Introduction:**

Use of three‐drug combinations is increasingly prevalent and associated with an increased risk of adverse drug events (ADEs). While real‐world data‐based pharmacovigilance and pharmacoepidemiology studies have derived knowledge on potential adverse three‐drug combinations, the relationship between doses of each drug in three‐drug combination exposure and the risk of ADEs remains unclear.

**Methods:**

We derived matched case–control datasets from US nationwide health insurance claims data for potential ADEs including acute kidney injury, acute myocardial infarction, gastrointestinal bleeding, hypoglycemia, and opioid‐related ADE. We used the conditional logistic regression model to investigate the relationship between the dose of three‐drug combination exposure and the risk of ADE. We used Benjamini and Hochberg's procedure to control the false discovery rate (FDR). We explored the relationship between the reduction of drug dose and the risk of ADE.

**Results:**

We identified over 500 potential adverse three‐drug combinations from approximately two million case–control pairs (all odds ratios ≥ 1.3 and FDR < 0.05). For the signals, compared with a high‐dose level of all three drugs, 74% of three‐drug combinations had a lower risk by decreasing the dose of one drug without any drug discontinuation (*p* value < 0.05).

**Conclusions:**

Certain three‐drug combinations are associated with an increased risk of ADE. Dose of exposure might be used to evaluate the risk of ADE for a majority of adverse three‐drug combinations.

**Plain Language Summary:**

Use of three‐drug combinations is increasingly prevalent and associated with an increased risk of adverse drug events (ADEs). We identified potential adverse three‐drug combinations from real‐world data, and revealed the corresponding relationships between doses and risks of ADEs. We find doses might be used to evaluate the risk of ADE for many adverse three‐drug combinations. Additionally, we find risk of many adverse three‐drug combinations might be decreased by reducing the dose of one drug without any drug discontinuation.

**Summary**

- Certain three‐drug combinations are associated with an increased risk of adverse drug events (ADEs).
- Dose might be used to evaluate the risk of many adverse three‐drug combinations.
- The risk of an adverse three‐drug combination might be decreased by reducing the dose of one drug without any drug discontinuation.
- Combination of opioid, other central nervous system drug, and antihypertensive drug might increase the risk of opioid‐related ADE.
- Combination of kidney‐eliminating drug and other nephrotoxic drugs might increase the risk of acute kidney injury.

## 1 Introduction

The concurrent use of three or more drugs (i.e., multi‐drug combinations) has become increasingly prevalent due to the growing demand for the management and/or prevention of multiple conditions. A recent US nationwide survey reported that 21% of the adult population and 66% of the older adult population are currently taking three or more medications [1]. As many chronic diseases require indefinite pharmacological management [2], the use of multi‐drug combinations is expected to increase further in the near future with extended longevity [3].

Exposure to multi‐drug combinations is associated with an increased risk of adverse drug events (ADE) [4, 5, 6, 7]. However, individuals with exposure to multi‐drug combinations are often underrepresented in clinical trials due to restrictive inclusion and exclusion criteria, limiting direct assessments of the relationship between multi‐drug combinations and the risk of ADE [8, 9, 10, 11]. Alternatively, large‐scale real‐world data (RWD; e.g., nationwide health insurance claims databases) include ample individuals with multi‐drug combination exposure [12]. RWD‐based pharmacovigilance studies have identified potential adverse three‐drug combinations related to bleeding [13, 14, 15], hypoglycemia [14], myopathy [16, 17, 18, 19], opioid‐induced ADE [14, 20], and unintentional traumatic injury [21, 22, 23]. As these studies are based on an exposed versus non‐exposed setting, they also reveal the comparative risk between potential adverse three‐drug combinations and the corresponding two‐drug sub‐combinations (e.g., A + B + C vs. A + B/A + C/B + C), which offers new opportunities to prevent and manage ADE by reducing drug exposure. Additionally, Shi et al. [14] conducted therapeutic class‐based data mining to identify alternative low‐risk multi‐drug combinations that share similar therapeutic classes with the corresponding high‐risk combinations. These alternative low‐risk multi‐drug combinations could mitigate the ADE burden without a significant compromise on the existing treatment regimen.

While these studies provide valuable knowledge on potential adverse three‐drug combinations, the relationship between the dose of drug exposure and the risk of ADE remains largely unexplored. As drug dose is a significant risk factor of ADE [24, 25, 26], we leverage US nationwide health insurance claims data to identify potential adverse three‐drug combinations and to explore the relationship between reductions in drug doses, drug exposure, and the risk of ADE. Specifically, we focus on five types of ADEs (acute kidney injury [AKI], acute myocardial infarction [AMI], gastrointestinal [GI] bleeding, opioid‐related ADE, and hypoglycemia), as these ADEs are common and potentially serious [27, 28, 29, 30], as well as likely caused by drug interaction and/or closely related to dose [31, 32, 33, 34, 35].

## 2 Materials and Methods

### 2.1 Data Source and Preprocessing

We used Optum's de‐identified Clinformatics Data Mart Database. The data were derived from US individuals enrolled in commercial health insurance and/or Medicare Advantage plans. The dataset included de‐identified information on enrollment, health encounter, diagnosis, and pharmacy claim. The Indiana University Institutional Review Boards (IRBs) designated this study as exempt.

**FIGURE 1:**
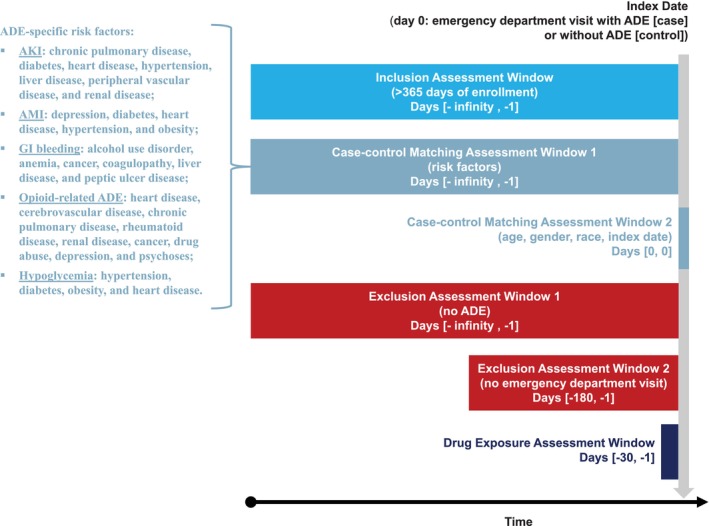
Illustration of the study design (ADE: adverse drug event, AKI: acute kidney injury, AMI: acute myocardial infarction, GI bleeding: gastrointestinal bleeding, opioid: opioid‐related ADE).

Figure 1 illustrated the study design [36]. We included emergency department (ED) visits from 2008 to 2021 that had > 365 days of enrollment and no ED visit within 180 days prior to the corresponding ED visit dates, regardless of whether the corresponding patients were discharged or hospitalized after ED visits. We used specific diagnosis codes to define five potential adverse drug event (ADE) outcomes including acute kidney injury (AKI) [37], acute myocardial infarction (AMI) [38], gastrointestinal (GI) bleeding [39], opioid‐related ADE [40], and hypoglycemia [41]. Additionally, we used R package comorbidity [42] to define risk factors for the potential ADEs. Specifically, the risk factors were (A) chronic pulmonary disease, diabetes, heart disease, hypertension, liver disease, peripheral vascular disease, and renal disease for AKI [43, 44]; (B) depression, diabetes, heart disease, hypertension, and obesity for AMI [45]; (C) alcohol use disorder, anemia, cancer, coagulopathy, liver disease, and peptic ulcer disease for GI bleeding [46, 47, 48, 49]; (D) heart disease, cerebrovascular disease, chronic pulmonary disease, rheumatoid disease, renal disease, cancer, drug abuse, depression, and psychoses for opioid‐related ADE [50], and (E) hypertension, diabetes, obesity, and heart disease for hypoglycemia [51]. Please see Supporting Information for additional details on variables.

### 2.2 Study Design and Analytical Datasets

We used the matched case–control design to derive five analytical datasets for the five potential ADE outcomes. Without loss of generality, we described the methods to derive one analytical dataset. As described in Figure 1, we defined cases as an individual's earliest known ED visit with the first and/or second diagnosis related to the potential ADE outcome. We excluded cases that had prior diagnoses related to the potential ADE outcome. We selected a control for each case matched on gender (exact), race (exact), risk factors (exact), age (±2 years), and year‐month of ED visit (±2 months), in which the matched controls also had no prior diagnosis related to the potential ADE outcome.

We defined the index dates as the ED visit dates for the cases and the matched controls. We assessed drug exposure data (e.g., drug name, quantity, dose [unit], and days of supply) within 30 days prior to the index dates (Figure 1). We used RxNorm and the data source's drug name table to identify drug ingredient names, and to process the top‐200 most frequent drug ingredient names in cases [52]. Please note that one drug ingredient name might have more than one unit (e.g., milligram and percent for a drug available in both tablet and topical forms). For any drug ingredient name–unit combination, we defined the average daily dose (ADS) as (dose [unit] × quantity)/days of supply; and defined a numerical variable for drug dose (i.e., 0, 1, 2), which 0 represented no exposure (i.e., ADS = 0), 1 represented low dose exposure (i.e., 0 < ADS ≤ median ADS in cases), and 2 represented high dose exposure (i.e., ADS > median ADS in cases).

We derived five analytical datasets. Each dataset included the following data elements: ADE outcome status (case = 1 and control = 0), matched pair ID, variables for drug doses for the top‐200 most frequent drug ingredient name‐unit combinations in cases.

### 2.3 Statistical Analyses

We used the following conditional logistic regression model (CLRM) to screen three‐drug combinations (Equation 1).

(1) $$\begin{array}{c} \text{logitProb}\left(\mathrm{ADE}=1\right)=\alpha +\beta_{1}X_{1}+\beta_{2}X_{2}+\beta_{3}X_{3}+\beta_{4}\left(X_{1}\times X_{2}\right)\\ \;+\beta_{5}\left(X_{1}\times X_{3}\right)+\beta_{6}\left(X_{2}\times X_{3}\right)+\beta_{7}\left(X_{1}\times X_{2}\times X_{3}\right) \end{array}$$

In Equation (1), Xs were variables for drug doses (i.e., X = 0, 1 or 2), α was the random effect for the matched pair, and β s were the log‐odds ratios (ORs).

As there were a large amount of three‐drug combinations, we tested three‐drug combinations that were frequent (*N* ≥ 500) in cases and related to the ADE outcome to maintain the statistical power. Specifically, for AKI, AMI and GI bleeding, the tested three‐drug combinations included at least one ADE‐related drug according to drug labels [53, 54]. For hypoglycemia and opioid‐related ADE, the tested three‐drug combinations included at least one antidiabetic drug and opioid, respectively. The drugs in the tested three‐drug combinations were summarized in Table S1. For each tested three‐drug combination, we estimated 7 parameters (i.e., β s) under the CLRM (Equation 1), seven *p* values for testing βs ≤ 0, and one *p* value for testing log‐OR ≤ 0 given $X_{1}$ = $X_{2}$= $X_{3}$ = 2 (i.e., no increased risk under the highest dose level; Equation 2). In other words, we computed eight *p* values for each three‐drug combination.

(2) $$H_{0}:2\;\beta_{1}+2\;\beta_{2}+2\;\beta_{3}+4\;\beta_{4}+4\;\beta_{5}+4\;\beta_{6}+8\;\beta_{7}\leq 0\;\mathrm{vs}.\neg H_{0}$$

We computed the Benjamini and Hochberg's false discovery rate (FDR) using all *p* values of all tested three‐drug combinations [55]. We selected the three‐drug combination associated with an increased risk under the highest dose level (e.g., Equation 2); as well as included three potential risky individual drug effects and/or significant interaction effect(s). Please see Supporting Information for additional details.

For signals, we investigated the relationship between reductions in dose (or exposure) and the risk of ADE. Under Equation (1), we estimated the ORs for the highest dose level (e.g., ($X_{1}$, $X_{2}$, $X_{3}$) = (2,2,2)) and other dose levels (e.g., ($X_{1}$, $X_{2}$, $X_{3}$) ≠ (2,2,2)). We compared the highest dose level to other dose levels under *z*‐test (Equation 3). We defined a significant risk reduction as *p* value < 0.05.

(3) $$\begin{array}{c} \text{Dose reduction for}\;\mathrm{one}\;\text{drug}:\\ \left(2,2,2\right)\;\mathrm{vs}.\left(2,2,1\right):\text{testing}\;\beta 3+2\beta 5+2\beta 6+4\;\beta 7\leq 0\;\mathrm{vs}.>0\\ \left(2,2,2\right)\;\mathrm{vs}.\left(2,2,1\right):\text{testing}\;\beta 2+2\beta 4+2\beta 6+4\;\beta 7\leq 0\;\mathrm{vs}.>0\\ \left(2,2,2\right)\;\mathrm{vs}.\left(1,2,2\right):\text{testing}\;\beta 1+2\beta 4+2\beta 5+4\;\beta 7\leq 0\;\mathrm{vs}.>0\\ \text{Dose reduction for}\;\mathrm{two}\;\text{drugs}:\\ \left(2,2,2\right)\;\mathrm{vs}.\left(2,1,1\right):\text{testing}\;\beta 2+\beta 3+2\beta 4+2\;\beta 5+3\beta 6+6\;\beta 7\leq 0\;\mathrm{vs}.>0\\ \left(2,2,2\right)\;\mathrm{vs}.\left(1,2,1\right):\text{testing}\;\beta 1+\beta 3+2\beta 4+3\;\beta 5+2\beta 6+6\;\beta 7\leq 0\;\mathrm{vs}.>0\\ \left(2,2,2\right)\;\mathrm{vs}.\left(1,1,2\right):\text{testing}\;\beta 1+\beta 2+3\beta 4+2\;\beta 5+2\beta 6+6\;\beta 7\leq 0\;\mathrm{vs}.>0\\ \text{Dose reduction for three drugs}:\\ \left(2,2,2\right)\;\mathrm{vs}.\left(1,1,1\right):\text{testing}\;\beta 1+\beta 2+\beta 3+3\beta 4+3\;\beta 5+3\beta 6+7\;\beta 7\leq 0\;\mathrm{vs}.>0\\ \text{Exposure reduction for}\;\mathrm{one}\;\text{drug}:\\ \left(2,2,2\right)\;\mathrm{vs}.\left(2,2,0\right):\text{testing}2\;\beta 3+4\beta 5+4\beta 6+8\;\beta 7\leq 0\;\mathrm{vs}.>0\\ \left(2,2,2\right)\;\mathrm{vs}.\left(2,0,2\right):\text{testing}2\;\beta 2+4\beta 4+4\beta 6+8\;\beta 7\leq 0\;\mathrm{vs}.>0\\ \left(2,2,2\right)\;\mathrm{vs}.\left(0,2,2\right):\text{testing}2\;\beta 1+4\beta 4+4\beta 5+8\;\beta 7\leq 0\;\mathrm{vs}.>0 \end{array}$$

We also conducted subgroup analyses in individuals with age ≥ 65 years. All analyses were conducted in R.

## 3 Results

### 3.1 Analytical Datasets and Signals of Adverse Three‐Drug Combinations

**FIGURE 2:**
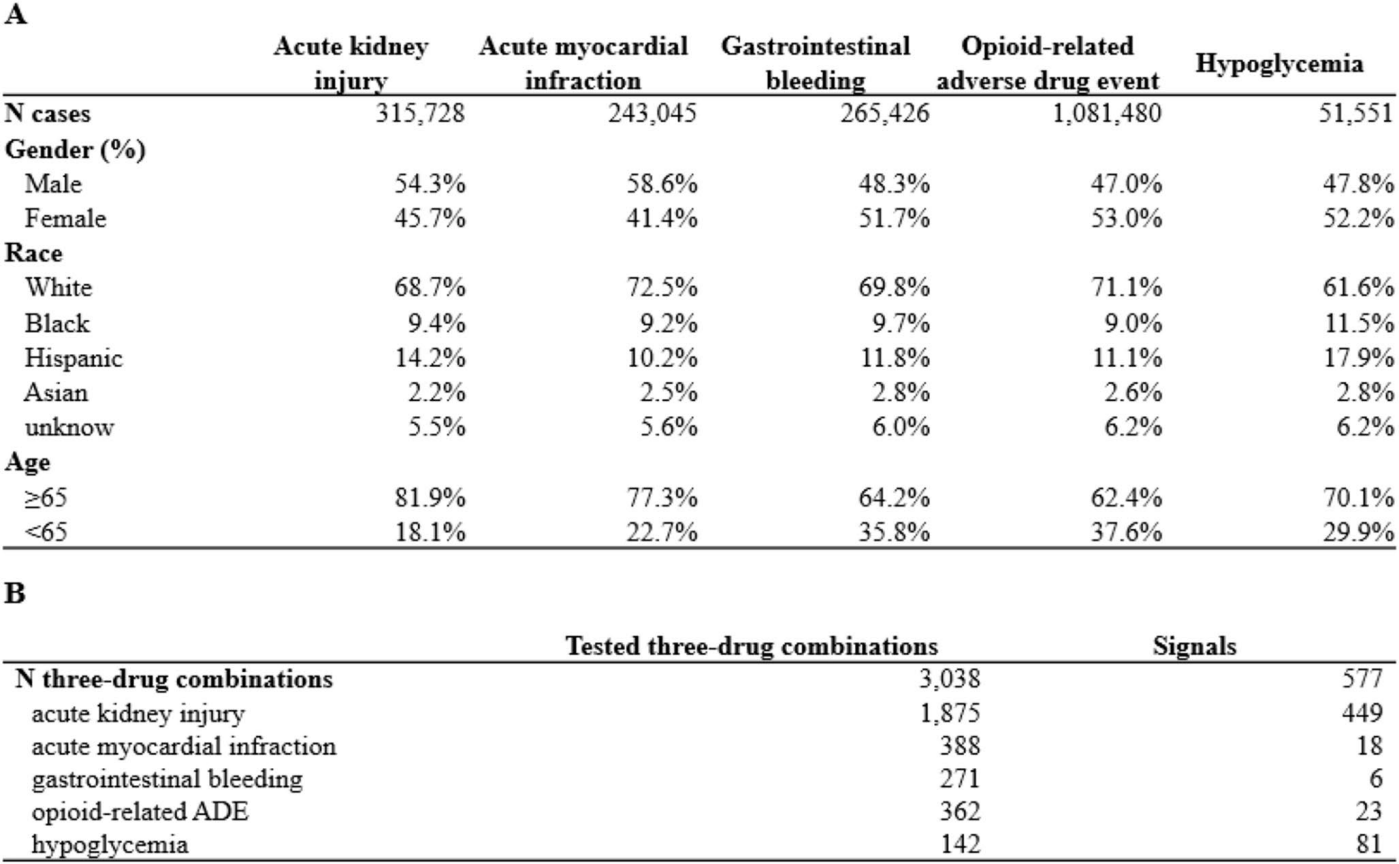
(A) Demographics of the study population; (B) signals of potential adverse three‐drug combinations.

Figure 2A presents the demographics of the final analytical datasets. The numbers of matched pairs were 315 728 pairs for AKI, 243 045 pairs for AMI, 265 426 pairs for GI bleeding, 1 081 480 pairs for opioid‐related ADEs, and 51 551 pairs for hypoglycemia. As shown in Figure 2A, most of the samples had White race and age ≥ 65 years, while 41%–53% of individuals were female. Figure 2B presents the frequencies of all tested three‐drug combinations and signals of potential adverse three‐drug combinations. We identified over 500 signals out of approximately 3000 tested three‐drug combinations (false discovery rate [FDR] < 0.05, Figure 2B), in which most of the signals were associated with an increased risk of acute kidney injury (AKI). We observed that the ORs of the three drug combinations were highly consistent between overall analyses and older adult (age ≥ 65 years) subpopulation analyses. Table S2 includes all signals and their odds ratios under the different dose levels.

### 3.2 Relationship Between Reduction in Drug Dose, Drug Exposure, and the Risk of ADE

**FIGURE 3:**
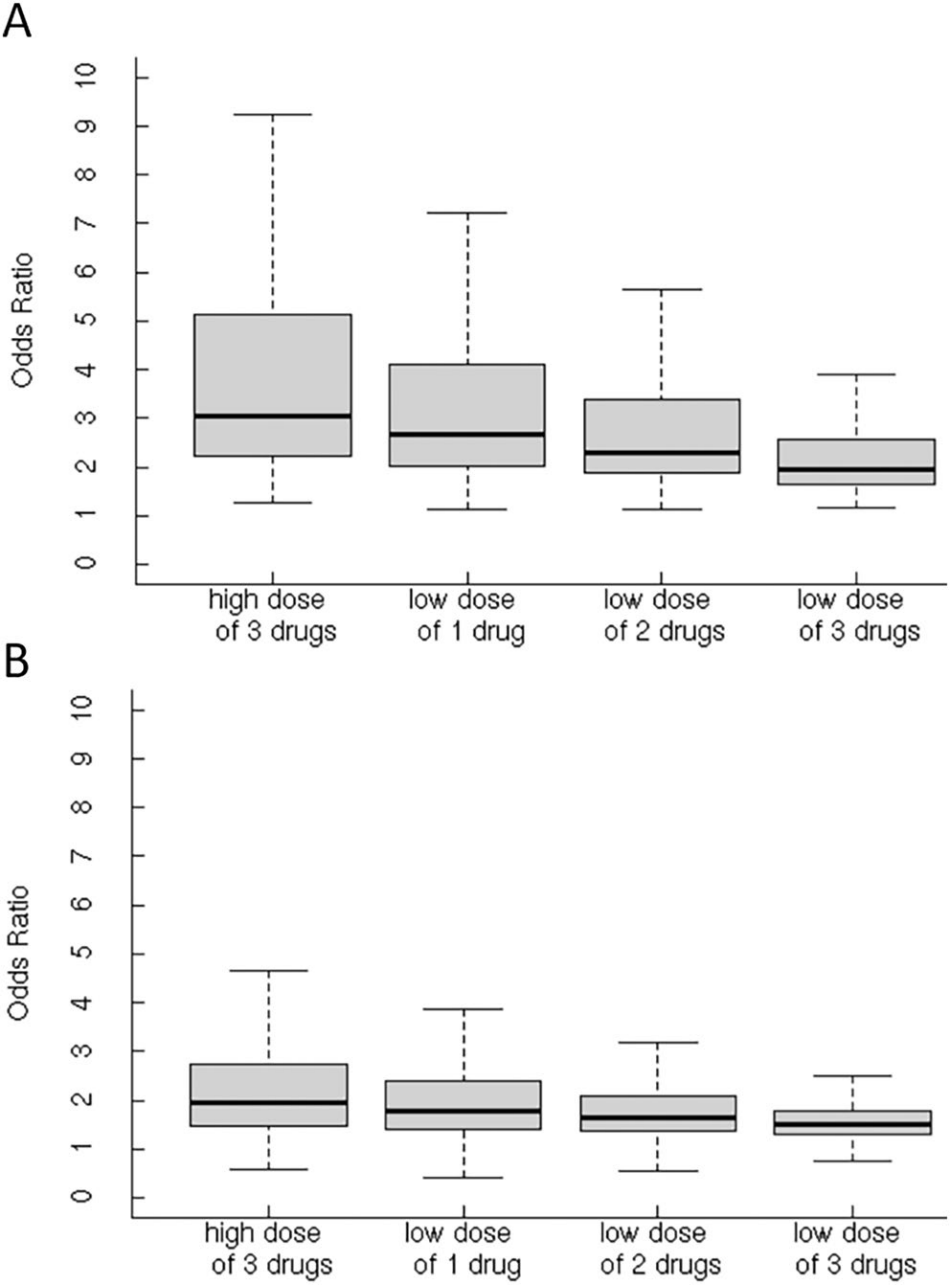
Distributions of odds ratios for the highest dose level, a lower dose for only one drug, lower doses for two drugs, and lower doses for three drugs; (A) signals; (B) all tested three‐drug combinations.

Figure 3 illustrates the estimated ORs for the three‐drug combinations with respect to the highest dose level (e.g., dose > threshold for 3 drugs), a lower dose for only one drug (e.g., dose > threshold for 2 drugs, and 0 < dose ≤ threshold for 1 drug), lower doses for two drugs (e.g., dose > threshold for 1 drug, and 0 < dose ≤ threshold for 2 drugs), and lower doses for three drugs (e.g., 0 < dose ≤ threshold for 3 drugs). For signals of potential adverse three‐drug combinations, the medians were 3.04 for the highest dose level, 2.66 for a lower dose for only one drug, 2.27 for lower doses for two drugs, and 1.93 for lower doses for three drugs (Figure 3A). For all tested three‐drug combinations, the medians were 1.94 for the highest dose level, 1.79 for a lower dose for only one drug, 1.63 for lower doses for two drugs, and 1.49 for lower doses for three drugs (Figure 3B).

**FIGURE 4:**
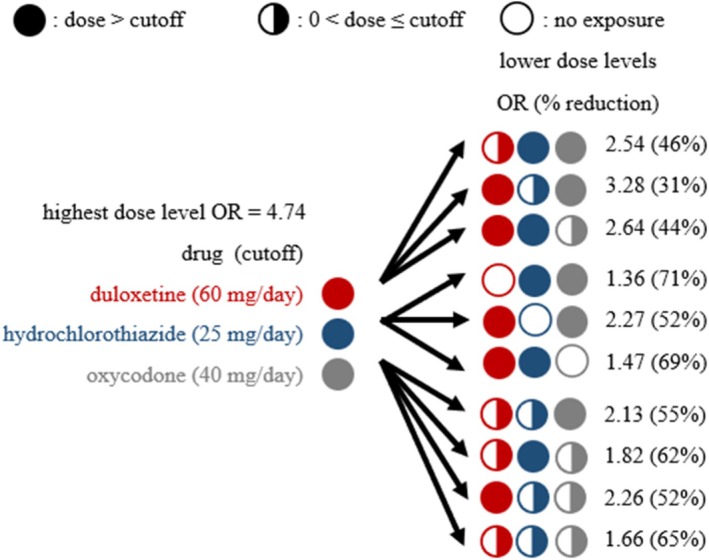
Relationship between dose of duloxetine‐hydrochlorothiazide‐oxycodone combination and odds ratios (ORs) of opioid‐related adverse drug events.

Figure 4 illustrates the potential relationship between dose levels of duloxetine‐hydrochlorothiazide‐oxycodone combination and ORs of opioid‐related ADE. Duloxetine‐hydrochlorothiazide‐oxycodone had OR = 4.74 under the highest dose level (e.g., duloxetine > 60 mg/day, hydrochlorothiazide > 25 mg/day, and oxycodone > 40 mg/day). Compared with the highest dose level (OR = 4.74), the decrements of OR were 31%–46% (ORs: 2.54–3.28) for reducing the dose of one drug, 52%–69% (ORs: 1.47–2.27) for reducing the exposure of one drug (i.e., discontinuation of one drug), and 52%–65% (ORs: 1.66–2.26) for reducing the dose of two or more drugs (all *p* values < 0.05).

**FIGURE 5:**
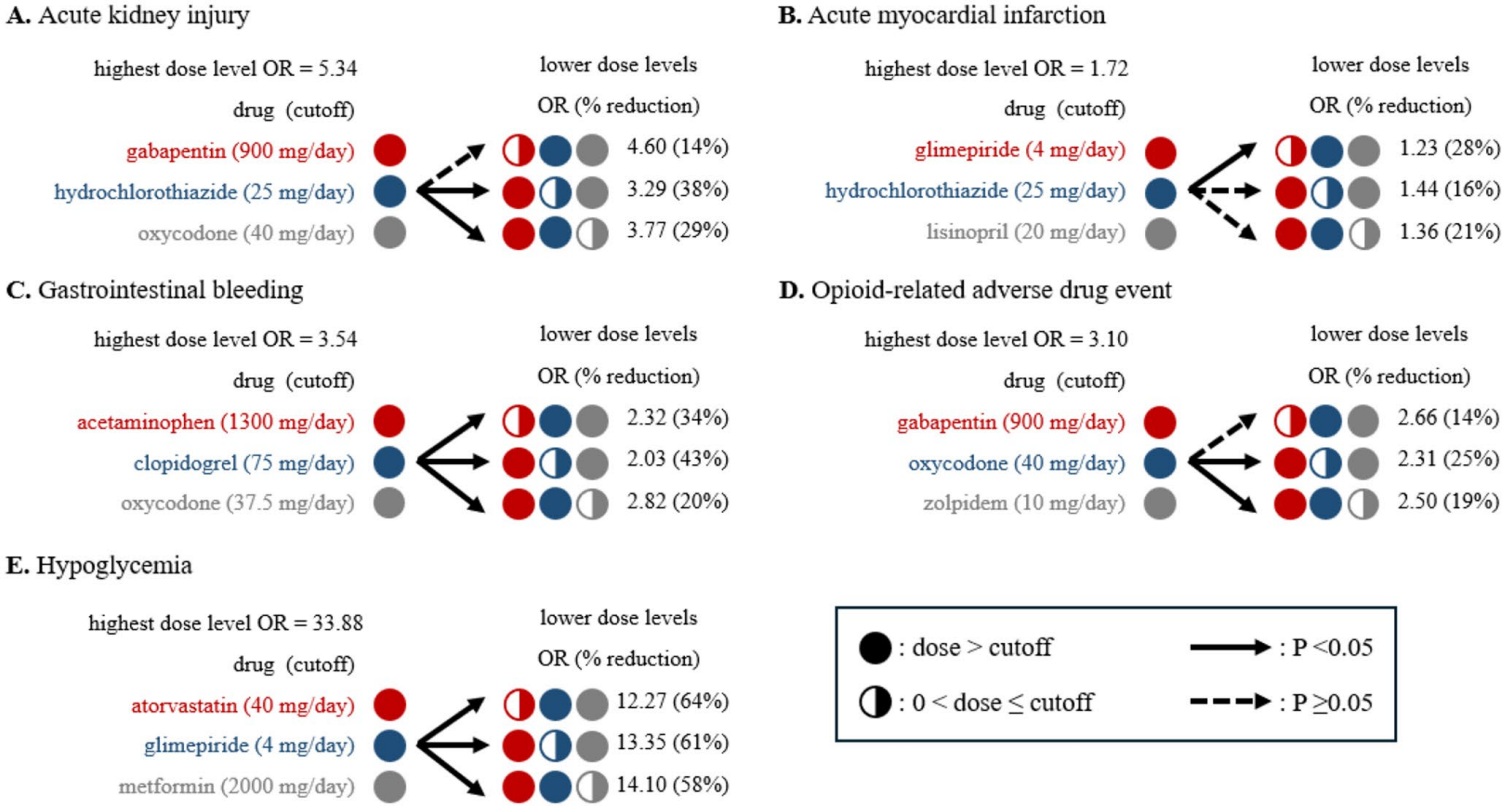
Relationship between dose of three‐drug combination and odds ratios (ORs) of adverse drug event (ADE); (A) Gabapentin‐hydrochlorothiazide‐oxycodone combination and acute kidney injury; (B) Glimepiride‐hydrochlorothiazide‐lisinopril combination and acute myocardial infarction; (C) Acetaminophen‐clopidogrel‐oxycodone combination and gastrointestinal bleeding; (D) Gabapentin‐oxycodone‐zolpidem combination and opioid‐related ADE; and (E) Atorvastatin‐glimepiride‐metformin combination and hypoglycemia.

Figure 5 presents five examples of potential dose‐related reduction in ADE risk, while the estimated ADE risks for all signals under the highest dose level a lower dose for only one drug can be found in https://riskroute.shinyapps.io/doseroute/. For AKI (Figure 5A), the gabapentin‐hydrochlorothiazide‐oxycodone combination had an estimated OR = 5.34 under the strongest dose level (e.g., gabapentin > 900 mg/day, hydrochlorothiazide > 25 mg/day, and oxycodone > 40 mg/day); and OR could be decreased by 29%–38% (ORs: 3.3–3.7) by reducing hydrochlorothiazide exposure to ≤ 25 mg/day or oxycodone exposure to ≤ 40 mg/day (all *p* values < 0.05). For AMI (Figure 5B), the glimepiride‐hydrochlorothiazide‐lisinopril combination had an estimated OR = 1.72 under the strongest dose level (e.g., glimepiride > 4 mg/day, hydrochlorothiazide > 25 mg/day, and lisinopril > 20 mg/day); and OR could be decreased by 28% (OR = 1.23) by reducing glimepiride exposure to ≤ 5 mg/day (*p* values < 0.05). For GI bleeding (Figure 5C), the acetaminophen‐clopidogrel‐oxycodone combination had an estimated OR = 3.54 under the strongest dose level (e.g., acetaminophen > 1300 mg/day, clopidogrel > 75 mg/day, and oxycodone > 3.75 mg/day); and OR could be decreased by 20%–43% (ORs: 2.03–2.82) by reducing any drug exposure to a lower dose (e.g., acetaminophen ≤ 1300 mg/day, clopidogrel ≤ 75 mg/day, or oxycodone ≤ 3.75 mg/day; all *p* values < 0.05). For opioid‐related ADE (Figure 4D), the gabapentin‐oxycodone‐zolpidem combination had OR = 3.10 under the strongest dose level (e.g., gabapentin > 900 mg/day, oxycodone > 40 mg/day, and zolpidem > 10 mg/day); and OR could be decreased by 19%–25% (ORs: 2.31–2.50) by reducing oxycodone exposure to ≤ 40 mg/day or zolpidem exposure to ≤ 10 mg/day (all *p* values < 0.05). For hypoglycemia (Figure 5E), the atorvastatin‐glimepiride‐metformin combination had an estimated OR = 33.88 under the strongest dose level (e.g., atorvastatin > 40 mg/day, glimepiride > 4 mg/day, and metformin > 2000 mg/day); and OR could be decreased by 58%–64% (ORs: 12.27–14.10) by reducing any drug exposure to a lower dose (e.g., atorvastatin ≤ 40 mg/day, glimepiride ≤ 4 mg/day, or metformin ≤ 2000 mg/day; all *p* values < 0.05).

## 4 Discussion

In this study, we identified potential adverse three‐drug combinations from a large‐scale US nationwide health insurance claims data. We investigated five types of adverse drug events (ADEs) including acute kidney injury (AKI), acute myocardial infarction (AMI), gastrointestinal (GI) bleeding, hypoglycemia, and opioid‐related ADE. We further evaluated the relationship between reductions of drug dose, drug exposure, and the risk of ADE. We found many adverse three‐drug combinations had a potentially lower risk under lower dose levels compared with a high dose level.

Our study generates real‐world evidence on potential three‐drug combination‐induced ADE. More importantly, our study, compared with previous studies, derives novel knowledge on the relationship between dose of drug exposure and risk of ADE, which might be used to mitigate the burden of ADE. For instance, the duloxetine‐hydrochlorothiazide‐oxycodone combination is associated with an increased risk of opioid‐related ADE (Figure 4). Besides oxycodone (an opioid), duloxetine (a central nervous system‐acting [CNS‐acting] drug for depression) is linked with sedation, and hydrochlorothiazide (a diuretic antihypertensive drug) is linked with dizziness [56, 57], both of which could adversely affect the risk and symptoms of opioid‐related ADE. In our study, compared with all three drugs at a high dose level (e.g., duloxetine > 60 mg/day, hydrochlorothiazide > 25 mg/day, and oxycodone > 40 mg/day), the odds ratio (OR) of ADE could potentially be decreased by 31%–46% by reducing the dose of one drug without any drug discontinuation, or potentially decreased by 52%–69% by reducing the dose of ≥ 2 drugs or discontinuing one drug (Figure 4). These exposure‐ and dose‐specific ORs could be used to guide dosage adjustment and drug discontinuation or initiation for mitigating the ADE burden. Additionally, other than concomitant CNS‐acting drugs, our results suggest common drugs linked with dizziness could also be a risk factor for opioid‐related ADE.

We would like to discuss several additional examples. Gabapentin‐hydrochlorothiazide‐oxycodone combination is associated with an increased risk of AKI (Figure 5A). Hydrochlorothiazide (a diuretic antihypertensive drug) could cause AKI and dehydration [58, 59]. Oxycodone (an opioid) could also cause AKI due to dehydration [60]. Gabapentin is eliminated in the kidney and has been reported to cause AKI [61]. In our study, compared with all three drugs at a high dose level (e.g., for gabapentin > 900 mg/day, hydrochlorothiazide > 25 mg/day, and oxycodone > 40 mg/day), the risk of ADE could be potentially decreased by 29%–38% by reducing the hydrochlorothiazide dose to ≤ 25 mg/day or the oxycodone dose to ≤ 40 mg/day. Our results suggested more considerations on drug dose and hydration status should be given for concomitant diuretic and opioid exposure, especially for patients with additional kidney‐eliminated drugs.

Moreover, as shown in Figure 5B–E, glimepiride‐hydrochlorothiazide‐lisinopril combination, acetaminophen‐clopidogrel‐oxycodone combination, gabapentin‐oxycodone‐zolpidem combination, and atorvastatin‐glimepiride‐metformin combination are associated with increased risks for different types of ADEs. All of these drug combinations include at least one drug that has been linked to the corresponding ADE [53, 54]. For instance, atorvastatin‐glimepiride‐metformin combination is associated with an increased risk of hypoglycemia. Atorvastatin could increase the risk of hypoglycemia at a higher dose [62], and glimepiride and metformin could cause hypoglycemia [53]. For these potential adverse three‐drug combinations, our results further reveal the relationship between dose of exposure and the risk of ADE. Compared with a high dose level of all three drugs, our study identifies the risk of ADE could be potentially decreased by 19%–64% by reducing the dose of one drug without any drug discontinuation. These findings suggest careful considerations on drug dose could mitigate the ADE burden. Additionally, more considerations on drug dose should be given for these potential adverse three‐drug combinations.

Our study also has several limitations related to real‐world data mining. First, as our analyses are based on US individuals with commercial health insurance or Medicare Advantage plans, our results might not be generalizable to other populations. Younger individuals with and without commercial health insurance may have differential health and socioeconomic statuses, as well as older individuals with and without Medicare Advantage plans. As both health status and socioeconomic status are linked to ADE risk [63], further studies are warranted to investigate the ADE risk in uninsured individuals, older adults without a Medicare Advantage plan, and socioeconomic status‐specific subpopulations. Second, we largely assume insurance records and claims are consistent with true clinical outcomes and drug exposure. However, ADE status and drug exposure could be misclassified in our analyses. We define a case as an emergency department (ED) visit with an ADE‐related diagnosis code in the first or second position. We focus on ED visits as the associations in an ED‐specific setting could better inform clinical decision making in an ED setting, especially given more than one million serious ADEs resulting in ED visits in the US each year [64]. Recent studies have found a high positive predictive value (PPV) for using diagnosis codes to define cardiovascular outcomes (e.g., PPVs ≥ 90% for the first diagnosis position, and ≥ 70% for both first and second diagnosis positions) [65, 66]. As a higher PPV is associated with reduced sensitivity, we define cases by using both the first and second diagnosis positions to balance PPV and sensitivity. Our study includes ED visits regardless of whether the corresponding patients are discharged or hospitalized after ED visits. Further studies are warranted to investigate the associations between drug exposure and hospitalizations following ED visits. Third, we collect drug exposure data within 30 days prior to the cases' and controls' index dates. The 30‐day exposure window has been used to identify clinically meaningful adverse drug combinations [15, 16, 17]. However, we cannot fully ensure the temporality between drug exposure and ADE onset. Our analyses do not differentiate patients with and without long‐term exposure and might have suboptimal power to detect drugs with a time of exposure‐dependent risk of ADE [67].

Our analyses are subject to unmeasured confounding effects, especially for confounding by indication and/or disease severity. The observed associations might be confounded by disease severity and/or treatment indication. For instance, high dose compared with low dose of atorvastatin‐glimepiride‐metformin exposure is associated with a higher risk of hypoglycemia (Figure 5E). Atorvastatin can manage hyperlipidemia, and glimepiride and metformin are diabetic drugs. Despite all of atorvastatin, glimepiride, and metformin being able to increase the risk of hypoglycemia at a higher dose level [53, 54, 62]; patients using higher doses of glimepiride and metformin may also have a greater severity of diabetes, which could increase the risk of hypoglycemia. Additionally, high dose compared with low dose of gabapentin‐hydrochlorothiazide‐oxycodone exposure is associated with a higher risk of AKI (Figure 5A). Gabapentin can manage epilepsy, hydrochlorothiazide can manage hypertension, and oxycodone can be used to manage cancer‐related pain. While all of gabapentin, hydrochlorothiazide, and oxycodone are linked to AKI [58, 59, 60, 61], their indications (e.g., epilepsy, hypertension, and cancer) could also increase the risk of AKI [43, 44, 68, 69]. Moreover, certain measurements (e.g., weight, creatinine, low‐density lipoprotein [LDL], hemoglobin A1c [HbA1c], etc.) could be linked with both the dose of drug exposure and the risk of ADE, while these variables are unavailable in our datasets. Additionally, weight could also relate to the dose of certain drugs and risks of ADEs. Given these limitations, our findings cannot be interpreted as causality. We expect future studies to validate our findings and further illuminate the mechanisms between the dose of three‐drug combination exposure and the risk of ADE. Clinical study or observational study using a perspective cohort design could be used to estimate the incidence and comparative risks between high‐dose and low‐dose exposure of three‐drug combinations. Additionally, a physiologically based pharmacokinetic model and quantitative system pharmacology model can validate the relationship between the dose of exposure and the risk of ADE.

Moreover, as the dose of drug exposure is related to both efficacy and toxicity, we expect future studies to evaluate the relationship between dose of exposure, risk of ADE, and efficacy. As the risk of potential adverse three‐drug combination could be reduced in multiple ways (Figures 4 and 5), we hypothesize the optimal dose of three‐drug combination can be derived to optimize the benefit–risk ratio. Finally, the median drug dose in the dataset is used as the cutoff for low versus high dose without consideration for clinical implications. This may limit clinical applicability, as our results were not reviewed for clinical applicability. We use median doses as thresholds under a rationale of high‐throughput data mining. By comparing our thresholds to the recommended doses defined in drugs.com, we find our thresholds could be meaningful. Using the duloxetine‐hydrochlorothiazide‐hydrocodone combination as an example (Figure 4), the median doses in our study and the recommended doses in drugs.com are: (i) duloxetine =60 mg/day in our study, and 40–120 mg/day for depression in drugs.com [70]; (ii) hydrochlorothiazide = 25 mg/day in our study, and 25–50 mg/day for hypertension in drugs.com [71]; and (iii) hydrocodone =40 mg/day in our study, and 20–80 mg/day for chronic pain in drugs.com [72]. Our methods can be applied to more clinically meaningful thresholds, while doing so requires clinical and pharmacological expertise with respect to a significant amount of drugs and adverse drug events (ADEs).

In conclusion, we identify potential adverse three‐drug combinations associated with an increased risk of ADE, and relationships between the dose of three‐drug combination exposure and the risk of ADE. Our analyses suggest careful consideration of drug dose could mitigate the ADE burden among patients using multiple drugs.

## Author Contributions

All authors participated in the design of the project, interpretation of results, and the critical writing and editing of the manuscript. Y.S., A.S., C.W.C., and P.Z. also participated in the data analyses.

## Ethics Statement

The Indiana University Institutional Review Boards (IRBs) designated this study as exempt.

## Conflicts of Interest

The authors declare no conflicts of interest.

## Supporting information


**Data S1:** Supporting Information.

**Table S1:** drugs in the tested three‐drug combinations.
**Table S2:** Signals of adverse three‐drug combinations. High level: dose > Cutoff; Low level: 0 < dose ≤ Cutoff; OR: odds ratio compared with no exposure; P: *p* value for testing a significant risk reduction compared with the highest dose under *z*‐test; 222: doses of all three drugs at the highest level; 122/212/221: reducing dose of Drug_1/Drug_2/Drug_3 to low level; 112/121/211: reducing doses of Drug_1 and Drug 2/Drug_1 and Drug_3/Drug_2 and Drug_3 to low level; 022/202/220: discontinue of Drug_1/Drug_2/Drug_3; 111: reducing doses of all three drugs to low level.

## Data Availability

Optum's de‐identified Clinformatics Data Mart Database is not publicly available (accessibility can be obtained from Optum: optum.com). Summary statistics generated during this study are included in this published article and its Supporting Information files.
