# Supplementary material for: Potential Drug Dose‐Specific Adverse Three‐Drug Combinations: A US Insurance Claims Data‐Based Study
Source: Pharmacoepidemiol Drug Saf. 2025 Aug 17;34(9):e70199. doi: 10.1002/pds.70199 (PMC12358767; doi:10.1002/pds.70199)
Supplement: Supplementary file 1 — Data S1: Supporting Information. [file PDS-34-e70199-s001.docx]

**Potential Drug Dose-specific Adverse Three-drug Combinations: A US Insurance Claims Data-based Study**

Y Shi,^1^ A Sun,^1^ CW Chiang,^2^ Y Yang,^1^ KM Hunold,^3^ J Xu,^1^ M Russo,^4^ J Caterino,^3,5^ MT Eadon,^6^ L Li^2^ J Su,^1^ M Donneyong,^7^ P Zhang^1 *^

1. Department of Biostatistics and Health Data Science, Indiana University, Indianapolis, IN, USA
2. Department of Biomedical Informatics, The Ohio State University, Columbus, OH, USA
3. Department of Emergency Medicine, The Ohio State University, Columbus, OH, USA
4. Department of Statistics, the Ohio State University, Columbus, OH, USA
5. Department of Internal Medicine, The Ohio State University, Columbus, OH, USA
6. Department of Medicine, Indiana University School of Medicine, Indianapolis, IN, USA.
7. College of Pharmacy, The Ohio State University, Columbus, OH, USA

* Correspondence author: Pengyue Zhang: zhangpe@iu.edu

**Supplementary Material S1:** Definition of variables.

We used the variable “Gender” and the variable “Race” in according to the data source. We defined Age according to the year of index date and the variable “Year of Birth” according to the data source. We defined adverse drug events and risk factors according to diagnosis codes from the data source, subject-matter-expert-derived algorithms,^1-5^ and R package comorbidity.^6^

**Supplementary Material S2:** Criteria for identifying signals.

For conditional logistic regression model

$\mathrm{logit} \mathrm{Prob}\left( ADE=1 \right)=\alpha+\beta_{1}X_{1}+\beta_{2}X_{2}+\beta_{3}X_{3}+\beta_{4}(X_{1}\times X_{2})+\beta_{5}(X_{1}\times X_{3})+\beta_{6}(X_{2}\times X_{3})+\beta_{7}(X_{1}\times X_{2}\times X_{3})$.

We defined signals as: FDR <0.05 for testing 2$\beta_{1}$+2$\beta_{2}$+2$\beta_{3}$+4$\beta_{4}$+4$\beta_{5}$+4$\beta_{6}$+8$\beta_{7}$ ≤0 (i.e., no increased risk under the highest dose level), and at least one of the following additional conditions should be true:

1. FDR <0.05 for testing $\beta_{i}$≤0 and $\beta_{i}$ >$\log2^{1/6}$ for all of $\beta_{1}$, $\beta_{2}$ and $\beta_{3}$; or
2. FDR <0.05 for testing $\beta_{i}$≤0 and $\beta_{i}$ >$\log2^{1/8}$ for at least two out of $\beta_{4}$, $\beta_{5}$ and $\beta_{6}$; or
3. FDR <0.05 for testing $\beta_{7}$≤0 and $\beta_{7}$ >$\log2^{1/8}$.

Specifically, additional condition 1 represented a combination of three adverse drugs, with the multiplicative OR >2 for the three drugs at the highest dose level; additional condition 2 represented at least two adverse drug-drug interactions, with the multiplicative OR >2 for those two adverse drug-drug interactions at the highest dose level; and additional condition 3 represented an adverse drug-drug-drug interaction, with the OR >2 at the highest dose level. The thresholds on $\beta$s could exclude three-drug combinations with a lower OR of ADE.

**References:**

1. Digmann R, Thomas A, Peppercorn S, et al. Use of Medicare Administrative Claims to Identify a Population at High Risk for Adverse Drug Events and Hospital Use for Quality Improvement. *J Manag Care Spec Pharm*. Mar 2019;25(3):402-410. doi:10.18553/jmcp.2019.25.3.402

2. Karter AJ, Warton EM, Moffet HH, et al. Revalidation of the Hypoglycemia Risk Stratification Tool Using ICD-10 Codes. *Diabetes Care*. Apr 2019;42(4):e58-e59. doi:10.2337/dc18-2154

3. Patel U, Hardy N, Smith D, et al. Validation of acute kidney injury cases in the mini-sentinel distributed database. Accessed 10/04/2022, <https://www.sentinelinitiative.org/sites/default/files/Drugs/Assessments/Mini-Sentinel_Validation-of-Acute-Kidney-Injury-Cases.pdf>

4. Saunders-Hastings P, Heong SW, Srichaikul J, et al. Acute myocardial infarction: Development and application of an ICD-10-CM-based algorithm to a large U.S. healthcare claims-based database. *PLoS One*. 2021;16(7):e0253580. doi:10.1371/journal.pone.0253580

5. U.S. Food and Drug Administration (FDA). Gastrointestinal Bleed Algorithm Defined in "Stroke, Gastrointestinal Bleeding, and Intracranial Hemorrhage following Apixaban or Warfarin Use in Patients with Non-Valvular Atrial Fibrillation: a Propensity Score Matched Analysis.". <https://www.sentinelinitiative.org/sites/default/files/surveillance-tools/validations-literature/GI_Bleed_apixaban_final_codelist.pdf>

6. Gasparini A. R Package ‘comorbidity’. 2022/05/23. <https://cran.r-project.org/web/packages/comorbidity/comorbidity.pdf>
